# Efficacy of Using Probiotics with Antagonistic Activity against Pathogens of Wound Infections: An Integrative Review of Literature

**DOI:** 10.1155/2019/7585486

**Published:** 2019-12-12

**Authors:** Sabina Fijan, Anita Frauwallner, Tomaž Langerholc, Bojan Krebs, Jessica A. ter Haar (née Younes), Adolf Heschl, Dušanka Mičetić Turk, Irena Rogelj

**Affiliations:** ^1^University of Maribor, Faculty of Health Sciences, Institute for Health and Nutrition, Žitna ulica 15, 2000 Maribor, Slovenia; ^2^Institut Allergosan, Pharmazeutische Produkte Forschungs-und Vertriebs GmbH, Gmeinstrasse 13, 8055 Graz, Austria; ^3^University of Maribor, Faculty of Agriculture and Life Sciences, Department of Microbiology, Biochemistry, Molecular Biology and Biotechnology, Pivola 10, 2311 Hoče, Slovenia; ^4^University Medical Centre Maribor, Department of Abdominal Surgery, Ljubljanska ulica 5, 2000 Maribor, Slovenia; ^5^Terhaar Consulting Inc., 36 Anvil Court, Richmond Hill, Ontario L4C 9G6, Canada; ^6^University of Maribor, Faculty of Medicine, Taborska ulica 8, 2000 Maribor, Slovenia; ^7^University of Ljubljana, Biotechnical Faculty, Institute of Dairy Science and Probiotics, Groblje 3, 1230 Domžale, Slovenia

## Abstract

The skin and its microbiota serve as physical barriers to prevent invasion of pathogens. Skin damage can be a consequence of illness, surgery, and burns. The most effective wound management strategy is to prevent infections, promote healing, and prevent excess scarring. It is well established that probiotics can aid in skin healing by stimulating the production of immune cells, and they also exhibit antagonistic effects against pathogens via competitive exclusion of pathogens. Our aim was to conduct a review of recent literature on the efficacy of using probiotics against pathogens that cause wound infections. In this integrative review, we searched through the literature published in the international following databases: PubMed, ScienceDirect, Web of Science, and Scopus using the search terms “probiotic” AND “wound infection.” During a comprehensive review and critique of the selected research, fourteen *in vitro* studies, 8 animal studies, and 19 clinical studies were found. Two of these *in vitro* studies also included animal studies, yielding a total of 39 articles for inclusion in the review. The most commonly used probiotics for all studies were well-known strains of the species *Lactobacillus plantarum*, *Lactobacillus casei*, *Lactobacillus acidophilus*, and *Lactobacillus rhamnosus.* All *in vitro* studies showed successful inhibition of chosen skin or wound pathogens by the selected probiotics. Within the animal studies on mice, rats, and rabbits, probiotics showed strong opportunities for counteracting wound infections. Most clinical studies showed slight or statistically significant lower incidence of surgical site infections, foot ulcer infection, or burn infections for patients using probiotics. Several of these studies also indicated a statistically significant wound healing effect for the probiotic groups. This review indicates that exogenous and oral application of probiotics has shown reduction in wound infections, especially when used as an adjuvant to antibiotic therapy, and therefore the potential use of probiotics in this field remains worthy of further studies, perhaps focused more on typical skin inhabitants as next-generation probiotics with high potential.

## 1. Introduction

According to the current definition, “probiotics are live microorganisms that, when administered in adequate amounts, confer a health effect on the host.” Both the Food and Agriculture Organization of the United Nations (FAO) and the World Health Organisation (WHO), as well as the International Scientific Association for Probiotics and Prebiotics (ISAPP), have developed and endorsed this definition of probiotics [1–3]. The most common probiotics are members of the *Lactobacillus* (e.g., including but not limited to strains of the following species: *Lactobacillus rhamnosus*, *Lactobacillus acidophilus*, *Lactobacillus plantarum*, *Lactobacillus casei*, and *Lactobacillus delbrueckii* subsp*. bulgaricus*) and *Bifidobacterium* genera (e.g., *Bifidobacterium infantis*, *Bifidobacterium animalis* subsp*. lactis*, and *Bifidobacterium longum*). Also, strains from other bacterial species (e.g., *Propionibacterium acidilactici*, *Lactococcus lactis*, *Leuconostoc mesenteroides*, *Bacillus subtilis*, *Enterococcus faecium*, *Streptococcus thermophilus*, and *Escherichia coli*) and certain yeasts (e.g., *Saccharomyces boulardii*) qualify as probiotics [4]. The best studied microbiome-management niche for probiotic action in the body is the gut.

With increasing knowledge about the essential role of gut microbiome in the human health, the gut microbiome is now considered an important ally, interacting with most human cells [5]. The discovery of links, or axes, for instance, the “gut-brain” and “gut-brain-skin,” has opened up new research dimensions. Besides mechanistic studies on fundamental topics (such as antimicrobial activity, competitive exclusion, immunomodulation, and strengthening of the intestinal epithelial barrier function), much research is focused on mechanisms of microbiome effects on the immune, the central nervous, and the endocrine systems [6–8]. Revolutionary discoveries about the importance of the human microbiome for human health have also accelerated further development of the probiotic sector. Scientific evidence of probiotic benefits on human health is continuously expanding, and there are enough data to justify investigation of probiotics for treatment or prevention of several disorders from antibiotic and *Clostridium difficile*-associated diarrhoea, irritable bowel syndrome, and inflammatory bowel disease to anxiety, depression, and wound healing [9–12].

The phrase “when administered,” in the definition of probiotics, can refer to the application of probiotics into the gut as well as on other sites (e.g., skin and vagina). Beneficial effects of probiotics have also been demonstrated in topical and *per os* use of probiotics in dental medicine, for women in urogenital infections, and in the respiratory tract. The use of probiotics is therefore widespread and one of the very promising areas is prevention and treatment of skin diseases. This review will systematically summarize the most recent *in vitro*, animal, and clinical studies on the antagonistic activity of probiotics against the pathogens of infected wounds.

### 1.1. Skin Microbiota

The skin is an important organ that represents the first line of defence against the external environment. Its main functions are to provide mechanical strength, regulate water and salt loss and protect the body from environmental damage, including that caused by microorganisms [13, 14]. Despite its tough physical characteristics, particularly in desiccated, nutrient-poor, acidic conditions, the skin is colonized by beneficial microorganisms that serve as an additional biophysical barrier to prevent the invasion of pathogens. When this barrier is disrupted or when the balance between commensals and pathogens is disturbed, skin diseases can appear. Using various state-of-the-art molecular and genetic-based methods, it was found that the skin microbiota is dominated by bacteria from the phyla Actinobacteria, Firmicutes, Proteobacteria, and Bacteroidetes; resident genera mainly include *Propionibacterium* spp., *Staphylococcus* spp., *Micrococcus* spp., *Corynebacterium* spp., and *Acinetobacter* spp. and the main representatives of the fungi being species of the genus *Malassezia* [15–18].

The diversity of skin microbiota among individuals depends on age, diet, gender, and environmental and geographical factors. However, the skin microbiota composition of healthy adults was found to be primarily dependent on the physiology of the skin site, with changes in the relative abundance of bacterial taxa. Sebaceous sites, for example, are dominated by lipophilic *Propionibacterium* species, whereas bacteria that thrive in humid environments, such as *Staphylococcus* and *Corynebacterium* spp., are preferentially abundant in moist areas, including the cubital fossa of the elbows and the underside of the feet. Overall, the skin harbours a heterogeneous community of microorganisms that each have distinct adaptations to survive on the skin [19].

### 1.2. Skin Damage and Wound Infections

Skin damage can be caused by a variety of different reasons such as trauma (including cuts, abrasions, chemical burns, fire burns, cold, heat, radiation, surgery), or as a consequence of underlying illnesses such as diabetes. The most effective wound management strategy is to prevent infections, promote healing, and prevent excess scarring [14]. The wound classification system categorizes all surgeries into four groups: clean, clean/contaminated, contaminated, and dirty [20]. Surgical site infections are currently one of the frequent types of nosocomial infections [21]. Chronically infected wounds, such as venous or arterial ulcers, diabetic foot ulcers, pressure sores, and nonhealing surgical wounds delay wound healing, have a significant impact on the patients' quality of life, represent a significant cause of morbidity and mortality, and result in enormous healthcare expenditures [14, 22–24]. Wound infections are most often caused by biofilm-forming bacteria such as *Staphylococcus aureus*, *Pseudomonas aeruginosa*, *Enterococcus faecalis*, *Acinetobacter baumannii*, *Escherichia coli*, *Klebsiella pneumoniae*, *Enterobacter* spp., *Peptostreptococcus* spp., etc., [25–32]. Biofilms are adherent communities of microorganisms that secrete a biochemical and physical matrix for protection, support, and survival; this matrix is a semipermeable barrier that limits diffusion of molecules that might otherwise gain access to planktonic microbes, such as quorum-sensing molecules and antibiotics. Biofilms impact chronic wound healing by delaying the inflammatory and maturation phases [14]. Different microbes are present during the beginning of a wound infection at neutral pH and after the wound becomes chronic when the pH becomes more alkaline and anaerobes are more likely to be present; causative agents of infections also differ according to wound type [26, 33].

### 1.3. Antibiotics: The Conventional Treatment for Wound Infections

Traditional therapy for infected wounds includes saline irrigation, debridement of necrotic tissues, and use of appropriate medications to reduce the microbial load such as local or systemic parenteral antibiotics and antiseptics [26]. However, an increasingly urgent problem is the resistance of microorganisms that commonly cause healthcare-associated infections to antimicrobial drugs [34].

Some experts claim that topical use of antibiotics or other medication is very important for the treatment of infected wounds (especially burns and chronic wounds) because the active substances of systemic antibiotics often do not reach the site of infection in sufficient quantities, namely, intravenous dosing of antibiotics is not as effective due to the reduction of microcirculation in the burned skin and the failure to eradicate biofilm infections. However, there are publications that state that topical use of antibiotics could more likely lead to the development of resistance than use of systemic antibiotics [14, 35]. Since it seems that antimicrobial resistance is transmitted even more frequently by topical application of antibiotics, the use of alternatives is imperative.

### 1.4. Probiotics as Alternatives to Antibiotics for Wound Infections

Antimicrobial resistance poses a serious global threat of growing concern to humans; therefore, alternatives to the topical skin antibiotics are of great interest. The Organisation for Economic Cooperation and Development (OECD) emphasizes that it is necessary to strengthen the scientific evidence of alternative therapies [36]. While some alternatives include inhibitors of antimicrobial resistance (e.g., alginate and polyamines), other chemical and biological agents with different mechanisms are currently being investigated: amino-benzimidazole, polyanionic substances, enzymes, potassium permanganate, antimicrobial peptides, metal ions (e.g., silver, bismuth, and copper), halogen ions (e.g., chlorine and iodine), chitosan, phototherapy, various antibodies, as well as bacteriophages and beneficial microorganisms, such as probiotics [37–41]. Interestingly enough, the OECD also states that probiotics are a promising alternative therapy to the topical use of antibiotics due to the increasing occurrence and transmission of antibiotic-resistant microorganisms.

**Figure 1:**
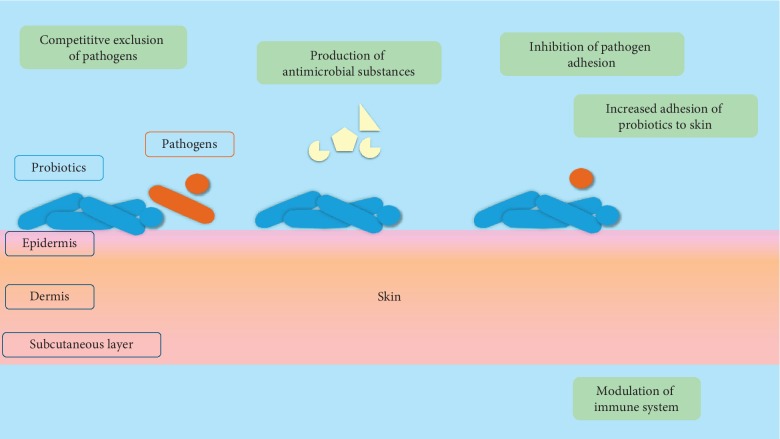
Proven and possible mechanisms of action of probiotics' antagonistic effects.

In the case of a disruption of the natural balance of skin microbiota, probiotics are known to have a positive effect on host health and skin healing through stimulating the production of immune cells and/or competitive exclusion of pathogens that cause skin infections [32, 42–44]. Probiotics release bioactive molecules that inhibit pathogen growth and interfere with the pathogens' quorum-sensing system. They furthermore coaggregate with pathogens, facilitating removal from the skin via peristaltic elimination, and can also displace them via high-affinity binding to epithelial cell receptors [45]. Some studies emphasize the use of cell-free metabolites, termed postbiotics, as safer and more effective than the use of live microbes [45], though this remains to be conclusively demonstrated. Other studies using cell lysates have proven to decrease parameters associated with skin inflammation by modulating the immune system both at local or systemic levels [46–48]. Probiotics promote wound healing, while acting at the epidermis and dermis levels, where they function as signalling receptors against pathogens and activate the production of beta-defensins, which enhance the immune capacity of the skin [49]. A description of the abovementioned proven and possible mechanisms of action of probiotics' antagonistic effects is shown in Figure 1.

Several studies demonstrating the positive effects of probiotics on wound healing have also been conducted *in vitro* or using animal models [42, 50–54]. There are clinical trials that prove efficacy of oral probiotics for various skin problems [22, 55] and even for lowering the rate of surgical site infections [56–58]. A recent meta-analysis [59] has also concluded that a reduction of surgical site infections following colorectal surgery was found for patients that were administered probiotics. The reported mechanisms mainly included immune modulation including: increase of production of TNF-*α* and IL-10 [59], systemic cellular immune response [56], modulation of the gene expression of SOCS3 [58], and pathogen inhibition [59].

Certain published studies also present the possibility of topical application of probiotics, probiotic supernatants or their metabolites for skin ulcers, burns, and other wounds. Most of these studies were carried out in burned animal models using mice, rats, pigs wherein the burn wounds were inoculated with selected pathogens (*P. aeruginosa* and *S. aureus*) and selected probiotics, and the reduction of the pathogen load was then observed [60, 61]. Reduction of pathogen load is a key parameter in establishing the healing trajectory [38, 62] and thus, arguably the most important effect of probiotics is their well-established antimicrobial effect against pathogens via the production of acids, bacteriocins or other antimicrobial molecules, and competitive exclusion. Exploring this antimicrobial effect of probiotics against wound pathogens was the main purpose of our review.

## 2. Materials and Methods

### 2.1. Search Strategy and Integrative Review Methodology

The present review includes a screening of the most recent studies on the antagonistic activity of probiotics against the pathogens of infected wounds and makes a comparison of *in vitro,* animal, and clinical studies. The mode of probiotic usage, namely, topical or systemic, is also noted.

**Figure 2:**
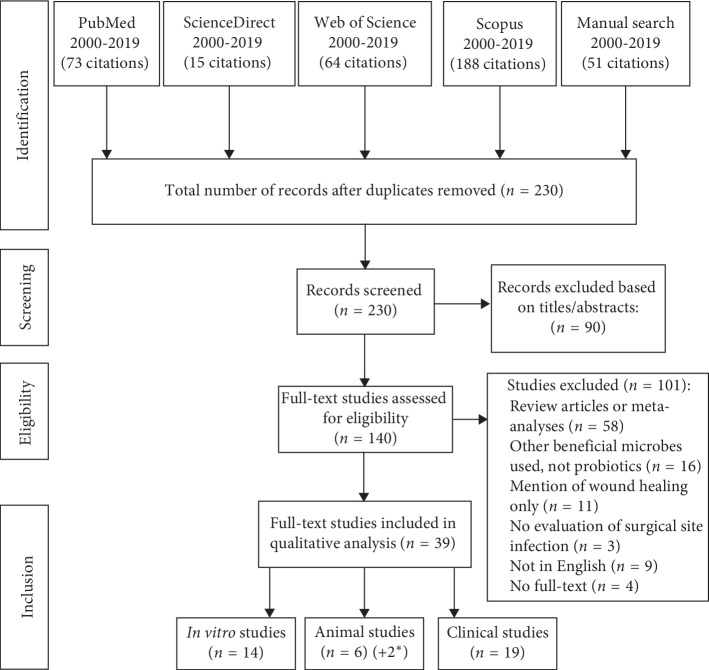
PRISMA flow diagram illustrating the process of literature screening, study selection, and reasons for exclusion. ^*∗*^Two studies reported an *in vitro* as well as one animal study in the same publication.

In order to obtain the most relevant selection of publications, the international databases PubMed, ScienceDirect, Web of Science, and Scopus were screened for studies using various keyword combinations: “probiotic” [MeSH] AND “wound infection,” “probiotic” AND “wound infection” [MeSH], “probiotics” AND “wound infections.” The PRISMA principles for data search were applied (http://www.prisma-statement.org/). Only English publications were included. Inclusion criteria were as follows: available full text and use of oral or topical probiotics for treating wound infections, live cultures associated with fermented foods, such as kefir and yogurt, were not included as these do not qualify as probiotics. Exclusion criteria were studies that only used probiotics for wound healing without mention of wound infections. Similar studies in articles' reference lists of reviews were also searched. A total of 391 articles were found (Figure 2). After removing duplicates, a total of 230 articles were screened and 90 were excluded based on title and abstract. 140 full texts were assessed for eligibility and 39 were included in the final analysis. These articles were then sorted by experimental design (*in vitro,* animal, and clinical studies) and entered in Tables 1–3; the mode of probiotic use is noted in Tables 2 and 3 as topical or systemic (oral). The literature search was concluded on the 24^th^ of June 2019 and coauthors SF and TL extracted the data from the searches.

As noted in Figure 2, the number of studies retrieved through database searching was very different for different databases despite the use of the same search parameters. This is probably due to the fact that each database contains different journals and publication sites. Several reviews were also found and their reference lists were screened with additional records noted in the manual search section.

## 3. Results

### 3.1. *In Vitro* Studies on the Use of Probiotics for Wound Infections

To date a large number of *in vitro* studies on the antimicrobial effects of probiotics against various pathogens exist [96].  Table 1 summarizes fourteen *in vitro* studies that include wound-specific pathogens and the potential use of probiotics to prevent their growth and development.

All fourteen studies in Table 1 showed efficient antagonistic effects of chosen probiotic strains against wound pathogens. The main techniques employed were different variations of the agar-well diffusion assay [63, 65, 70, 71, 74] and the coculturing method [61, 65, 67, 72, 73], and *S. aureus*, *P. aeruginosa*, *E. coli*, and *A. baumannii* were the most commonly investigated pathogens. The most commonly used probiotics were various strains of *L. plantarum* (six studies), *L. acidophilus* (four studies), and *L. reuteri* (four studies). Four studies included supernatants or extracts produced by probiotic strains [67, 69, 71, 74], whilst the other studies used live probiotic cultures. Eight studies included various monospecies probiotics, whilst six studies included multispecies probiotics [64, 66, 70, 71, 73, 75]. Two studies from Table 1 [61, 68] also included animal model experiments and are additionally noted in Table 2.

Although two additional studies [97, 98] showed that strains of *L. acidophilus* and *L. casei* exhibited efficient antagonistic effects against wound pathogens using the well diffusion method, they are not included in Table 1, since the lactobacilli were isolated from buffalo milk curd and yogurt and are outside of the probiotic framework since their clinical effects on health have not been demonstrated [3]. Significant antagonistic effects of lactic acid bacteria against wound pathogens (*P. aeruginosa*, *C. albicans*, *S. aureus*, and *E. coli*) [99] and *Aerococcus viridians* against wounds infected with *S. aureus* and *Salmonella enterica* serovar Typhimurium [100] were also published in two studies in 2000 and 1998, respectively; however, the articles were not in English with no information on the methodology in the English abstract and were therefore also excluded.

### 3.2. Animal Studies on Use of Probiotics for Wound Infections

All animal studies on the antimicrobial effects of probiotics against skin pathogens, deliberately added on burns or wounds on animals, can be found in Table 2. A total of eight animal studies met the inclusion criteria, two of which are mentioned in Table 1 [61, 68].

The studies investigated burn wounds, ischemic wounds, and skin lesions. Three studies each used mouse [61, 68, 78] and rat models [76, 80, 81] and two studies used rabbit models [77, 79]. Local application of probiotics was used for six studies and only two studies included local injections [61, 78] of probiotics. Oral probiotic administration was not utilized in any study. The most frequently used probiotic was *L. plantarum* ATCC 10241 (six studies). All animal studies resulted in an efficient antagonistic effect of probiotics against wound pathogens, mainly *P. aeruginosa*, followed by *S. aureus*.

Three studies [101–103], not included in Table 2, used kefir and kefir extracts against various pathogens applying *in vitro* methods and burn rat models with positive outcomes of effective antibacterial effects and wound healing. Although the kefir microbiota contain a diverse group of live beneficial microorganisms, it is not classified as a probiotic *per se* as it is not well defined in terms of strain composition, health effects, and stability [3]; therefore, these articles could not be added to Table 2. Another publication by Al-Mathkhury and coworkers [104] was also not included in Table 2; it showed that *L. plantarum*, *L. bulgaricus,* and *L. acidophilus*, isolated from yogurt, vinegar, and the human vagina, respectively, also exhibited antimicrobial properties when added to mice' wounds previously infected with *S. aureus* or *P. aeruginosa*. However, again according to the panel of the ISAPP [3], live cultures (traditionally associated with fermented foods), for which there is no evidence of a health benefit, are not probiotics; therefore, this study could not be included. Another animal model publication [105] reported the effectiveness of a *Bacillus* strain against *Streptococcus pyogenes* infection of surgical wounds on rats; however, only the abstract was available in English and therefore was excluded from Table 2. Another excluded study [106] successfully used skin commensal *Staphylococcus epidermidis* on a mice model with infected skin. Of note, some articles also recommend the use of bacteriophages for treatment of infectious wounds [107–109], which are currently not included in the definition of probiotics.

### 3.3. Clinical Studies on the Use of Probiotics for Wound Infections

In demonstrating the impact of probiotics on general health as well as in connection with the use for wound infections, the most important studies are randomized double-blinded clinical trials with a representative sample. We found a total of nineteen studies (eighteen clinical trials and one case study) that met the inclusion criteria and these are noted in Table 3. The clinical trials of various surgeries mainly included routinely used antibiotic therapy that varied between groups. The potential influence of probiotics on the duration of antibiotic therapy is shown in Table 3. The methodology of the studies was also assessed using a Critical Appraisal Skills Program (CASP) checklist tool [110] for randomised controlled trials (Table 4) and case-control studies (Table 5).

Topical application of probiotics was used only in two studies, one on infected foot ulcers and the other on burns [22, 86]. There were two additional studies [92, 93] and one case study [55] on burn injuries with oral use of probiotics. All these studies resulted in a decreased pathogenic load with probiotic administration.

The remaining fourteen studies listed in Table 3 used oral probiotic administration and were conducted on surgical patients with surgical site wounds as well as underlying diseases or conditions such as cancer, transplantation, etc. The main reason for using probiotics in these clinical trials was to enhance wound healing and prevent systemic and surgical site infections after surgery. The patients of these studies also received routine antibiotic prophylaxis (mainly one dose intravenous before surgery). The studies were only included in Table 3 if surgical site infections were recorded. Seven studies concerned colorectal cancer surgery [57, 58, 87, 89, 91, 94, 95], three studies were for liver surgery [84, 88, 90], two studies for biliary cancer surgery [56, 83], and one each for abdominal surgery [82] and pancreaticoduodenectomy [85]. All of these studies except one [57] noted a tendency of lower incidences of surgical site infections in the probiotics group; only two noted a statistically significant difference of surgical site infections in the probiotics group [58, 91] vs. the placebo group. On other hand, one study noted a statistically significant higher incidence of surgical site infections in the probiotic group versus the antibiotic group, but no statistically significant difference in the control group [57]. Several studies noted a statistically significant lower incidence of systemic infections, bacteraemia, urinary tract infections, pneumonia, and peritonitis and hence better healing, however not in all cases. Eight studies assessing surgical site infections used synbiotics [56, 82–85, 88, 90, 94] and six studies used probiotics [57, 58, 87, 89, 91, 95].

The clinical study of patients undergoing pancreaticoduodenectomy [111] also showed that perioperative probiotics reduced postoperative infectious complications; however, it was not included in Table 3 as only an abstract was available. The study by McNaught and coauthors [112] was not included in Table 3 as surgical site infections were only mentioned in the initial part of the study before using antibiotics for all patients. Studies on the application of probiotics in the treatment of patients with nonhealing purulent-inflammatory wounds [113] and patients with colorectal surgery [114] were also found; however, the articles were not available in English and could not be further assessed.

As the aim of this integrative review was to find all possible studies using different methods on the use of probiotics against wound pathogens, none of the clinical studies demonstrating probiotics efficacy against wound infections were omitted even if the scores of the CASP checklist included several negative answers as noted in Tables 4 and 5.

### 3.4. Most Commonly Used Probiotics for Wound Infections

Table 6 includes the total set of probiotic species from Tables 1–3 that have been used against common wound pathogens.

Regardless of the study type (*in vitro*, animal model, or clinical study), by far, the most commonly used probiotics were various strains of *L. plantarum*, followed by *L. cassei*, *L. acidophilus*, *L. rhamnosus*, *L. fermentum*, *B. breve*, and *B. longum*. Confirming what was aforementioned, it is obvious that the genus *Lactobacillus* was the most commonly used. All other genera, including *Bifidobacteria* and other lactic acid bacteria, such as *Enterococcus* spp., *Pediococcus* spp., and *Leuconostoc* spp., were minimally used and mainly as components of multispecies probiotics. There were also a limited amount of studies using bacteria from the *Bacillus* genera and the yeast *S. boulardii*. Only one study used a probiotic strain of the skin bacterium *Propionibacterium acnes*.

## 4. Discussion

Many centuries ago, even before mankind knew microbes existed and before the use of antiseptics and antibiotics, fermented milk was applied to wounds to improve healing and prevent infection [49]. The use of bacteria to fight bacteria is therefore an old concept, especially with respect to the skin. According to Sprunt & Leidy [115], the first attempted replacement of one microorganism by another was done by Cantini in 1885 who claimed to replace *Mycobacterium tuberculosis* (then named *Bacillus tuberculosis*) in the lungs with another harmless organism. Metchnikoff, who is named the father of probiotics, also mentioned this principle in the early 1900s, as did Nissle, who, in 1916, used an *E. coli* strain for the treatment of various intestinal disorders [105, 116]. Today, however, this represents a major shift in the paradigm of the current doctrine of wound treatment as well as the traditional teaching of “germ theory” where the idea of using bacteria to fight bacteria is not intuitive [21, 49]. It has been 15 years since the publication of the review by Howard and coauthors on the possible use of probiotics in surgical wound infections; however, not much has changed with regard to the traditional therapy of wound infections and more clinical evidence is still necessary for a paradigm shift in this area [117].

Several reviews on the use of probiotics for wounds in general or for specific conditions have been published [60, 118–120]; however, to the best of our knowledge, no systemic review specifically on the influence of probiotics against wound pathogens has been conducted. There are also several reviews on the general effect of probiotics on healing after surgery [121–123]; however, our focus was on the antagonistic effect of probiotics. The review by Besselink and coauthors [121] on the potential role of probiotics in the prevention of complications in surgical patients in general also concluded that probiotics show promising results in several clinical trials, although the review was not focused on surgical site infections, but rather on bacterial translocation due to gut dysfunction at the mucosal barrier. The same conclusions were drawn in the review on the use of probiotics for patients undergoing abdominal surgery [122] and colorectal resection for cancer [123].

The most important studies that demonstrate the impact of probiotics on health in general are randomized, double-blinded, placebo-controlled clinical trials with a representative sample and proper study design, and these trials represent the final phase of traditional product development trajectory, which can be conducted only after the successful completion of preceding robust preclinical studies. Reliance on *in vitro* data or animal models alone is not sufficient as these data may not directly correlate to clinical evidence and limited data presented in human studies [124]. However, certain traits and characteristics of candidate probiotics for use in wound infections must be tested by *in vitro* methods such as adhesion and inhibition of pathogen adhesion to human keratin as well as the production of antimicrobial substances [52, 71].

All investigated *in vitro* studies on the antagonistic activity of chosen topical probiotics against common wound pathogens yielded the same general result, namely, an effective inhibition of the growth of wound pathogens. However, these studies are only the first step, as they do not take into account the influence of the host and system matrix, more specifically, the layers of the skin. The most commonly studied probiotic bacterial taxon (*Lactobacillus*) does not primarily belong to the skin microbiota [125]. It should also be noted that probiotics are not expected to colonize the skin for extended periods of time, an often-misunderstood concept for successful probiotic action. Rather, they are chosen due to their scientifically proven antagonistic effect against the conventional nosocomial and gastrointestinal pathogens, which are strikingly similar to the most common skin pathogens [126]. An appropriate alternative for studying interactions between probiotics and pathogens, which is becoming more established, is the *in vitro* use of cell lines that mimic the original environment of the organism in the form of a biological matrix [127, 128]. For *in vitro* studies of the human skin function, the most popular cell line has been HaCaT, a spontaneously mutated keratinocyte cell line from immortalized adult skin [129]. There is also some published literature on the use of models to simulate wound healing [130, 131], but there is still no published literature on the use of probiotics with them. Another possibility is the use of the nematode's *Caenorhabditis elegans* epidermis as a model skin [132, 133]. There is even an international patent for microspheres from gelatin as a carrier for probiotic *Lactobacillus* spp. for treating skin wounds or lesions [134].

Our search yielded eight animal model studies using probiotics against wound pathogens, three on mice, and two each on rats and rabbits. All studies confirmed an effective antagonistic effect of probiotics towards pathogens, mainly various strains of *L. plantarum*, regardless of whether the wound was an infected burn or cut wound. Six animal studies used topical application of probiotics on the wounds, and two studies used near-site injections and all studies resulted in successful reduction of the two most common skin pathogens, *S. aureus* and *P. aeruginosa*. Furthermore, all studies concluded that the investigated probiotic could be applied to human wound infections. In terms of wound healing experiments, mice and rats are the most commonly used animal models. It must be stressed, however, that these animals have a thinner epidermis and dermis compared to humans, thus bringing into question suitability of such an animal model. On the other hand, experiments on large animals, such as pigs, whose skin has been regarded as the closest surrogate to human skin with regard to similarities in structure and healing, have a disadvantage of extensive costs, handling, and lack of genetic manipulability [131, 135].

Certain probiotics have been reported to form robust biofilms *in vitro* and shown to attach to various host biofilm sites; these include *L. casei*, *L. rhamnosus*, *L. plantarum*, *L. reuteri*, *L. acidophilus*, *B. bifidum*, and *B. breve* [136–141]. Although probiotics form similar biofilm modalities as pathogens, research and evaluation of these biofilms has only occurred in recent years and not necessarily on the skin [43]. It is also a question of whether these *in vitro* biofilms are representative of the *in vivo* situation.

Only two clinical studies used topical application of *L. plantarum* ATCC 1024 on infected wounds: in one case, a burn wound [22] and in the other case, chronic foot ulcers [86]. In the clinical study on burns, it was found that the topical application of the *L. plantarum* ATCC 1024 on burns was as effective against pathogens as topical application of silver ions [22]. In the second clinical study on diabetic patients with chronic ulcers, topical application of *L. plantarum* ATCC 1024, besides achieving a statistically significant decrease of pathogen load after 10 compared to day 1 with topical probiotic treatment, also improved healing; higher production of IL-8 and a reduction in the number of infected ulcers was furthermore achieved [86].

Fourteen clinical studies in our review were conducted on patients with various abdominal surgeries (colorectal cancer surgery, liver transplantation, abdominal surgery, and others). The main reason for using probiotics in these clinical trials was to enhance wound healing and prevent systemic and other infections after surgery in general, one aspect being surgical site infections, although not the main focus.

An important aspect of the use of probiotics in wound infections is the concomitant use of probiotics with antibiotic treatments. The evidence reviewed in this manuscript seems to suggest a potential role for adjuvant probiotic therapy in surgery. Some studies demonstrated statistically lower duration of antibiotic therapy [56, 82, 84, 85, 90]; others showed a nonsignificant trend towards reduced antibiotic duration [83, 95], while the rest showed no difference in duration of antibiotic therapy in probiotics or synbiotics groups. The main antibiotics were and various third-generation cephalosporin antibiotics; certain probiotic strains are naturally resistant to certain cephalosporins, or metronidazole [88, 142], whilst other reports indicate that various *bifidobacteria* strains are susceptible to metronidazole [142], suggesting that coadministration of probiotics within antibiotic therapy must be further guided by data regarding the antimicrobial resistance of the probiotic strains. Combined therapy with antibiotics and probiotics can have a beneficial and stabilizing effect on the intestinal metabolic homeostasis [143], but further research is necessary.

All clinical studies except one reported a lower incidence of surgical site infections which resulted either in a statistically lower [58, 91], or trending but not statistically significant, surgical site infection rate after probiotic administration. In one noted exception [57], all patients received a single dose of intravenous preoperative, second-generation antibiotic, whereas the antibiotic group also received kanamycin sulphate and metronidazole before the operation as a chemical bowel preparation; thus, even the initial conditions were not uniform compared to the probiotics and control groups which received no antibiotic therapy after surgery. These results show that probiotics could be used as adjuvant therapy before and after surgery, but not instead of antibiotic therapy. However, this does not mean that all probiotic clinical studies before surgery necessarily result in benefit of intervention [144].

The main reported pathogens found in surgical site wound infections were *S. aureus*, *P. aeruginosa*, A*. baumannii*, *E. coli*, *E. cloacae*, *E. faecium*, *or E. faecalis*, which coincides with the findings of other research of probiotic adjuvant therapy [13]. In the investigated clinical studies, the most commonly used probiotics were strains of *L. plantarum*, *L. casei*, and *L. acidophilus*. These three species of the genus *Lactobacillus* have well-known and well-studied strain-specific abilities. Selected strains of *L. acidophilus* and *L. casei* aid in effectively reducing *C. difficile* infections [145] and *H. pylori* infections. Selected strains of lactobacilli aid in epithelium restitution during wound repair and can inhibit colonization of other species in the wound [146]. It seems that lactobacilli successfully amplify the antimicrobial effect against pathogens in wounds, but may not specifically enhance the immune system of the host, which was in fact the main rationale behind studying probiotics in these clinical trials. Perhaps different combinations of strain-specific probiotics [3] could be more successful in reducing wound infections through synergistic and complimentary mechanisms of action. It is well established that orally consumed probiotics aid in supporting the body's immune response, and therefore the systemic action of probiotics to promote wound healing is another important strategy. Some studies [82, 147] have found that postoperative consumption of probiotics (mainly *L. plantarum* 299) *per os* improves immune response, reduces the number of postoperative infections, and reduces hospitalization time and the amount of prescribed antibiotics. All of these studies conclude that postoperative endpoints should continue to be thoroughly investigated, and two studies went on to highlight the great potential of topical use of probiotics to protect the wound [15, 17].

Eight of the fourteen clinical trials assessing surgical site infections from our literature search included oral synbiotics for patients undergoing surgery [56, 82–85, 88, 90, 94]; therefore, one could argue that it is not possible to determine whether the positive influence can be attributed to the individual components, the probiotics, or the prebiotics. Although it is well known that prebiotics are utilized by probiotics [148], when comparing these eight clinical trials and the other six clinical trials [57, 58, 87, 89, 91, 95] on surgical patients that received only probiotics, differences or better results for the studies that utilized synbiotics compared to the studies that utilized only probiotics were not observed. As noted by some [149], certain studies lacked placebo control groups [56] or were not double-blinded [91, 94], thus limiting the ability to describe the efficacy of the administered probiotics. This was also confirmed in the review by Gurusamy and coauthors [150] on the methods for preventing wound complications after liver transplantation. The authors concluded that there were no statistically significant differences in the probiotics/synbiotics group in graft rejections, intensive unit stay, hospital stay, and mortality; however, it was found that a statistically significant lower proportion of these patients in the probiotics group developed infective complications, thus confirming at least one positive effect after probiotic administration.

## 5. Conclusion

Although this review is directed at the antimicrobial role of probiotics in combating wound infections and has shown promising results as possible alternatives or adjuvant therapies, the problem is still more complex. In order to achieve optimal wound healing, it is necessary to address in parallel additional factors regarding the patient's general health or the wound's physical environment and the body's immune response [23, 151]. Despite the fact that it is known that wound healing is impaired by wound infection, the exact role of probiotics in delayed wound healing remains controversial due to discrepancy in clinical results [14, 64, 152]. However, an impressive number of studies as noted in this review have shown that exogenous and oral application of probiotics together with antibiotics before and after surgery has shown reduction in wound site infections and shorter duration of antibiotic therapy. In addition, topical application of probiotics for burn infections and chronic ulcers decreased the pathogen load. Therefore, the potential use of probiotics for wound infections remains worthy of some more intense future study [153]. Further studies could also be warranted for topical application of probiotics, perhaps focused more on typical skin inhabitants as topical probiotics with high potential.

## Figures and Tables

**Table 1:** *In vitro* studies on the antimicrobial effect of probiotics against wound pathogens.

First author, year	Pathogen species	Probiotic(s)	Method	Outcome	Potential use for humans
Valdez, 2005 [61]^#^	*Pseudomonas aeruginosa*	*Lactobacillus plantarum* ATCC 10241	Coculturing	Greatest inhibitory activity with whole culture, somewhat lower inhibition with acid filtrate	Local treatment of burn infections

Jones, 2010 [63]	*Escherichia coli*, *Staphylococcus aureus*, *P. aeruginosa,* MRSA, *Trichophyton mentagrophytes*, *Trichophyton rubrum*	*Lactobacillus fermentum* NCIMB 7230	Agar-well diffusion method	Nitric oxide-producing patch with probiotic, killed all common bacterial and fungal wound pathogens	Antimicrobial applications for infected wounds

Thomas, 2011 [64]	*S. aureus*, *P. aeruginosa*, *Candida albicans*	*Lactobacillus reuteri* ATCC 55730, *Lactobacillus casei*^*∗*^, *L. plantarum*^*∗*^	Triphasic PLUS wound model	Different efficiency of probiotics against different pathogens	Potential benefit of wound colonization with single or mixed probiotics

Varma, 2011 [65]	*S. aureus*, *P. aeruginosa*	*L. fermentum* ^*∗*^	Coculturing and well diffusion assay	Both pathogens were successfully inhibited	Inhibition of common wound pathogens

Prince, 2012 [66]	*S. aureus*	*L. reuteri* ATCC 55730, *Lactobacillus rhamnosus* AC413	Cell culture	Inhibited adherence of pathogen to keratinocytes	Topical prophylaxis in preventing skin infection

Ramos, 2012, [67]	*P. aeruginosa*	*L. plantarum* ATCC 10241 supernatant	Culturing pathogen with probiotic supernatant	Antipathogenic properties	Infected chronic wounds

Shu, 2013 [68]^#^	MRSA USA300	*Propionibacterium acnes* ATCC6919 extract	Agar spot with propionic acid	Effective inhibition of pathogen	Skin health

Mohammedsaed, 2014 [69]	*S. aureus*	*Lactobacillus rhamnosus* GG lysate and spent culture supernatant	Normal human epidermal keratinocyte suspension	Inhibition of pathogen growth and reduction of pathogen adhesion	Damaged skin

Al-Malkey, 2017 [70]	*P. aeruginosa*	*L. rhamnosus* GG, *L. acidophilus*^*∗*^	Well diffusion assay	Antimicrobial effect of probiotic bacteriocins against burn wound pathogen	Preventing hospital-acquired infections

Lopez, 2017 [71]	*E. coli*, *P. aeruginosa*, *S. aureus*, *Propionibacterium acnes*, *Propionibacterium aeruginosa*	Supernatants of *Lactobacillus delbrueckii* DSMZ 20081, *Bifidobacterium animalis* CHR Hansen Bb 12, *L. acidophilus* La-5, L-10, L-26, *Bifidobacterium lactis* B-94, *Bifidobacterium longum* DSMZ 20088, *L. plantarum* 226v, *Lactobacillus brevis* D-24, *Lactobacillus salivarius* DSMZ 20555, *L. casei* DSMZ 20021, CHR Hansen 01, 431	Well diffusion assay; attachment assay	Prevent biofilm formation and exhibited antimicrobial activity against skin pathogens	Topical application for skin dysbiosis

Chan, 2018 [72]	*Enterobacter hormaechei*, *Klebsiella pneumoniae*, *Acinetobacter baumannii*	*L. reuteri* SD2112	Coculturing	Differential gene response, pili formation, cell attachment	Polymicrobial wound infections

Li, 2018 [73]	*P. aeruginosa*, *S. aureus*	*L. acidophilus* CL1285, *L. casei* LBC80R, *L. rhamnosus* CLR2	Probiotic encapsulation and coculturing with pathogens	Encapsulated probiotics in combination with antibiotics results in complete eradication of pathogens	For topical coadministration with antibiotics

Onbas, 2018 [74]	*P. aeruginosa,* MRSA	*L. plantarum* F-10 (a promising probiotic strain), cell-free extract	Agar-well diffusion assay, biofilm formation, coaggregation, quorum-sensing	Antimicrobial, anti-biofilm, antiquorum-sensing activity	Against skin infections

Soleymanzaheh, 2018 [75]	*P. aeruginosa*	*L. reuteri* DSM17938, *L. acidophilus* DSM, *Bacillus coagulans* DSM1, *L. plantarum* 299v, DSM9843, *Bifidobacterium bifidum* DSM20456	Disc diffusion method	Some probiotics and antibiotics exhibited synergistic effects; other combinations exhibited antagonistic effect	Possible use of certain probiotics with certain antibiotics to create synergistic effects on wound healing.

^#^Study also included animal model. ^*∗*^Strain not specified.

**Table 2:** Animal model studies on the antimicrobial effects of probiotics against wound pathogens.

First author, year	Animal	Wound type	Pathogen species	Probiotic(s)	Method	Outcome	Potential use for humans
Valdez, 2005 [61]^#^	Mice	Burn wound	*Pseudomonas aeruginosa*	*Lactobacillus plantarum* ATCC 10241	Injection into burned area (10^5^ cfu/mL injected into burned area on days 3, 4, 5, 7 and 9)	Inhibitory effect against pathogen and wound improvement	Local treatment of burn infections

Brachkova, 2011 [76]	Rats	Burn wound	*P. aeruginosa*	*L. plantarum* ATCC 8014	Topical application on burned area (single dose 10^8^ cfu/mL)	Reduction of pathogen load in wound	Intervention for prevention of multiresistant pathogen infection in burns

Jones, 2012 [77]	Rabbits	Ischemic wound	*Staphylococcus aureus*	*Lactobacillus fermentum* 7230	Local application of patches designed with lyophilized probiotic microbeads (single dose of 10^6^ cfu/mL)	Improvement of probiotic treated wounds through nitric oxide production	Chronic wounds

Shu, 2013 [68]^#^	Mice	Skin lesion	MRSA USA300	*Propionibacterium acnes* ATCC6919	Local topical application of *Propionibacterium* (10^5^ cfu/mL for 17 days)	Decrease in cfu of pathogen	Skin wound and skin health

Argenta, 2016 [78]	Mice	Burn-sepsis wound	*P. aeruginosa*	*L. plantarum* ATCC 10241	Subeschar injection (10^9^ cfu/mL daily for 5 days)	Lower mortality rate and inhibition of pathogen in remote organs	Management of complicated burn injury

Satish, 2017 [79]	Rabbits	Burn-sepsis wound	*P. aeruginosa*	*L. plantarum* ATCC 10241	Local application (single dose of 3 × 10^8^ cfu)	Curtailed severity and length of infection as well as reduced scarring	Counteracting burn wound infection and alleviate scarring

Ong, 2019 [80]	Rats	Full-thickness wound	*S. aureus*	*L. plantarum* USM8613	Single local application of 10% (v/v) protein-rich fraction of cell-free supernatant with paraffin	Higher reduction of pathogen with probiotic and enhanced wound healing	Inhibition of wound pathogens

Surmeli, 2019 [81]	Rats	Third-degree scald burn	MRSA ATCC 43300	*L. plantarum* ATCC 10241	Local application (single dose of 1 × 10^6^ cfu/mL)	Protective role when applied before pathogen	Promising role in prevention and treatment of wound infections

^*∗*^ *In vitro* study included in Table 3. MRSA: methicillin-resistant *S. aureus*.

**Table 3:** Eighteen clinical studies and one case study on the antimicrobial effects of probiotics against wound pathogens.

First author, year	Study type noted in paper	Wound type	Patients PR/CO	Wound pathogen	Probiotic/total cfu per day	Antibiotic treatment	Probiotics treatment	Wound infections (%) PR/CO	Outcome
Rayes, 2002 [82]	Prospective, randomized	Abdominal surgery	30/30	Streptococci	*L. plantarum* 299^*∗∗∗*^, (2 × 10^9^ cfu) with fibres; heat killed bacteria as placebo	For all patients before surgery. After surgery in cases of expected or proven infection.	Oral (for 4 days after surgery)	0%/3%	Lower incidence of surgical site infections, however not statistically significant. Placebo group received antibiotic therapy significantly longer than group with probiotics and fibres.

Kanazawa, 2005 [83]	Randomized, controlled	Biliary cancer surgery	21/23	*S. aureus*, *E. faecalis*, *Enterococcus faecium*, *Enterobacter cloacae*	*Lactobacillus casei* Shirota, *Bifidobacterium breve* Yakult/(2 × 10^8^ cfu)^*∗∗∗*^	For all patients before surgery. After surgery in cases of expected or proven infection.	Oral (for 14 days after surgery)	14.3%/26.1%	Significantly lower incidence of overall infections in the synbiotics group. Lower, but not statistically significant, incidence of wound infections. Slightly lower duration of postoperative antibiotic therapy for synbiotics group.

Rayes, 2005 [84]	Randomized, double-blind	Liver transplant surgery	33/33	*S. aureus*	*Pediococcus pentosaceus* LMG P-20608, *Leuconostoc mesenteroides* LMG P-20607, *Lactobacillus paracasei* subsp*. paracasei* LMG P-17806*; L. plantarum* LMG P-20606 (10^10^ cfu)^*∗∗∗*^	For all patients before surgery. After surgery in case of bacterial infection.	Oral (starting on the day of surgery for two weeks)	0%/3%	Lower incidence of wound infection for probiotics with prebiotics group, significantly lower overall postoperative bacterial infections in the same group. Significantly lower duration of antibiotic therapy in synbiotics group.

Sugawara 2006 [56]	Randomized, controlled	Biliary cancer surgery	40–41^#^/0	Not mentioned	*L. casei* Shirota, *B. breve* Yakult/(before surgery 5 × 10^10^ cfu)^*∗∗∗*^; (after surgery 2 × 10^8^ cfu) ^*∗∗∗*^	For all patients before surgery. After surgery if needed.	Oral (14 days before and 1^st^ day after surgery for 14 days) or after surgery for 14 days	4.8%–15%/NR	Lower incidence of wound infection for probiotics with prebiotics perioperative and postoperative treatment, statistically significantly lower overall postoperative infections and duration of antibiotic therapy for the same group.

Rayes, 2007 [85]	Randomized, double-blind	Pancreaticoduodenectomy	40/40	Not mentioned specifically for wound infections	*P. pentosaceus* LMG P-20608, *L. mesenteroides* LMG P-20607, *L. paracasei* subsp*. paracasei* LMG P-17806*; L. plantarum* LMG P-20606 (10^10^ cfu)^*∗∗∗*^	For all patients before surgery. After surgery in case of bacterial infection.	Oral (starting on the day after surgery for 8 days)	10%/15%	Lower incidence of wound infection for probiotics with prebiotics group, statistically significantly lower overall postoperative infections and duration of antibiotic therapy in synbiotics group for same group.

Peral, 2009 [22]	Prospective	Second and third-degree burns	38/42	*S. aureus*, *Pseudomonas aeruginosa*, *S. epidermidis*, *E. cloacae*, *Klebsiella pneumoniae*, *E. faecalis*	*L. plantarum* ATCC 10241 (10^5^ cfu)	Antibiotics are not routinely administered for burn patient due to their cost and of the high degree of antibiotic resistance	Daily topical application for 10 days	NA	Topical probiotic treatment of 2^nd^ degree burn patients was as effective as silver sulphadiazine in control group in decreasing pathogen load.

Peral, 2010 [86]	Prospective	Chronic infected leg ulcers	34^##^/0	*S. aureus*, *P. aeruginosa*, *S. epidermidis*, *E. cloacae*, *K. pneumoniae*, *E. faecalis*	*L. plantarum* ATCC 10241 (10^5^ cfu)	Not administered due to extreme resistance in chronic wounds.	Daily topical application, 10 days	NA	Statistically significant decrease of pathogen load after 10 days (*P* < 0.001) compared to day 1 with topical probiotic treatment. However, non-probiotic group was not applied.

Liu, 2011 [87]	Randomized, double-blind, placebo-controlled	Colorectal cancer surgery	50/50	Not mentioned	*L. plantarum* CGMCC 1258, *L. acidophilus* LA-11, *Bifidobacterium longum* LB-88/(2.6 × 10^14^ cfu)	For all patients before surgery. After surgery if needed.	Oral 16 days (6 days preoperatively and 10 days postoperatively)	6%/10%	Low incision site infection rate, however not statistically significant. No statistically significant difference in length of antibiotic therapy.

Usami, 2011 [88]	2-arm, randomized, controlled	Hepatic surgery	32/29	MRSA	*L. casei* Shirota, *B. breve* Yakult/(6 × 10^8^ cfu)^*∗∗∗*^	For all patients before surgery. After surgery if needed.	Oral (14 days before operation and 11 days allowed food intake)	0%/6.9%	No infectious complications after surgery in probiotic group resulting in a statistically significant difference (*P* < 0.05)

Thomson, 2012 [55]	Case study	Deep-dermal and full-thickness burn patient	1	XDR *P. aeruginosa*	*L. casei* Shirota (6.5 × 10^9^ cfu)	Patient received 10 different antibiotics during her hospital stay.	Oral (for 2 weeks after infection which occurred 5 months after burn)	NA	Pathogen from wound changed from multidrug resistant to multidrug sensitive strain, thus implying effective intervention

Zhang, 2012 [89]	Randomized, double-blind, placebo-controlled	Colorectal cancer surgery	30/30	Not mentioned	*B. longum* ^*∗*^, *Lactobacillus acidophilus*^*∗*^, *Enterococcus faecalis*^*∗*^ (3 × 10^8^ cfu)	For all patients before surgery and after surgery for 3 to 5 days. If infection occurred an additional regimen was given.	Oral (3 to 5 days before surgery)	3.3%/13.3%	Lower surgical site infection rate for probiotics group, however not statistically significant

Zhang, 2013 [90]	Prospective, randomized	Liver transplant surgery	34/33	*Enterococci* spp, *Enterobacter* spp, *Escherichia coli*	*L. acidophilus* LA-14, *L. plantarum* LP-115, *Bifidobacterium lactis* BBL-04, *L. casei* LC-11, *Lactobacillus rhamnosus* LR-32, *Lactobacillus brevis* LBr-35/(2.75 × 10^10^ cfu)^*∗∗∗*^	Antibiotic therapy post operation, if necessary.	Oral (at least 7 days after oral fluid tolerance after operation)	5.9%/15.2%	Incidence of postoperative infections was lower for probiotic with fibre group compared to fibre only. Significantly shorter duration of antibiotic therapy in group with probiotics and fibre.

Sadahiro, 2014 [57]	Prospective, randomized, double-blinded, controlled	Colorectal cancer surgery	99/95^*∗∗*^	*E. coli*, *S. aureus*, *P. aeruginosa*, *S. epidermidis*, *E. faecalis*, *Bacteroides fragilis*, *Serratia marcescens*	*Bifidobacterium bifidum* ^*∗*^ (3.3 × 10^9^ cfu)	For all patients before surgery. After surgery only for antibiotic group.	Oral (7 days before and 5 to 10 days after operation)	6.1%/17.9%	The probiotics group had a slightly higher rate of surgical site infections vs. control group. The probiotics group had a statistically significant higher rate of surgical site infections than the antibiotic group.

Aisu, 2015 [91]	Clinical trial	Colorectal cancer surgery	75/81	Not mentioned	*E. faecalis* T110, *Clostridium butyricum* TO-A, *Bacillus mesentericus* TO-A (no information on concentration)	For all patients before surgery and after surgery for two days.	Oral (15 days prior surgery, restarted the same day the patient started drinking water after surgery	6.7%/19.8%	Significant lower surgical superficial incisional site infection (*P*=0.016)

Kotzampassi, 2015 [58]	Randomized, double-blinded, placebo-controlled	Colorectal cancer surgery	84/80	*Acinetobacter baumannii*, *P. aeruginosa,* MRSA	*L. acidophilus* LA-5, *L. plantarum*^*∗*^, *B. lactis* BB-12, *Saccharomyces boulardii*^*∗*^/(5.5 × 10^9^ cfu)	Not mentioned	Oral (1 day prior to operation and 14 days after surgery)	7.1%/20.0%	Statistically significant decrease in surgical site infections (*P*=0.02)

Mayes, 2015 [92]	Randomized, blinded	Burn injury	10/10	Not specified	*L. rhamnosus* GG (1.5 × 10^10^ cfu)	Days of receiving antibiotic medications recorded	Oral (start within 10 days after burn and until 95% wound closure)	NA	Trend of less requirement for antifungal agents (*P*=0.03) in probiotic group. No significant difference in number of days of antibiotic therapy

El-Ghazely, 2016 [93]	Randomized, double-blinded, controlled	Burn	20/20	Not specified	*Lactobacillus fermentum* ^*∗*^ and *Lactobacillus delbrueckii*^*∗*^/(2.0 × 10^9^ cfu)	Not mentioned	Oral – during hospital stay	35%/60%	Trend towards decrease in infection incidence (*P*=0.113).

Kotmatsu, 2016 [94]	Single-centre, randomized controlled	Colorectal resection	168/194	Not specified	*L. casei* Shirota, *B. breve* Yakult/(4.0 × 10^10^ cfu)^*∗∗∗*^	For all patients before surgery.	Oral (7–11 days before surgery and reintroduced at 2–7 postoperative days)	17.3%/22.7%	Trend towards lower surgical site infection rate for synbiotic group, however not statistically significant (*P*=0.2). Study was not blinded and no placebo product was used.

Yang, 2016 [95]	Randomized, double-blinded	Colorectal cancer surgery	30/30	Not specified	*B. longum* ^*∗*^, *L. acidophilus*^*∗*^, *E. faecalis*^*∗*^/(3.0 × 10^7^ cfu)	For all patients before surgery. After surgery if needed.	Oral 12 days (5 before, 7 after surgery)	3.3%/3.3%	No statistically significant differences in wound infection rates. Slightly lower postoperative duration of antibiotic therapy for probiotics group.

PR/CO, probiotic vs. control group; NR, not reported specifically for wound infection; NA, not applicable; ^*∗*^strain not specified; ^*∗∗*^additional antibiotic group in study (100 patients), ^#^40 patients received postoperative synbiotics treatment and 41 patients received both preoperative and postoperative synbiotic treatment, ^*∗∗∗*^probiotic used together with prebiotic or fibre, ^##^14 diabetic patients and 20 nondiabetic patients; MRSA: methicillin-resistant *S. aureus*, XDR: multidrug resistant.

**Table 4:** CASP quality assessment checklist of included clinical trials using the CASP checklist for randomised controlled trials.

First author, year	Section A						Section B		Section C		
	1	2	3	4	5	6	7	8	9	10	11
Rayes, 2002 [82]	Yes	Yes	Yes	Yes	Yes	No	Small	Partial	Yes	Yes	Yes
Kanazawa, 2005 [83]	Yes	Yes	cannot tell	cannot tell	Yes	Yes	Some	Partial	Yes	Yes	Yes
Rayes, 2005 [84]	Yes	Yes	Yes	Yes	Yes	Yes	Small	Partial	Yes	Yes	Yes
Sugawara 2006 [56]	Yes	Yes	Yes	cannot tell	Yes	NA^*∗*^	NA^*∗*^	Partial	Yes	No	Yes
Rayes, 2007 [85]	Yes	Yes	Yes	Yes	Yes	Yes	Small	Partial	Yes	Yes	Yes
Peral, 2009 [22]	Yes	cannot tell	Yes	cannot tell	Yes	cannot tell	Large	Partial	Yes	Yes	Yes
Peral, 2010 [86]	Yes	No	Yes	No	No	NA^*∗*^	NA^*∗*^	Partial	Yes	No	Yes
Liu, 2011 [87]	Yes	Yes	Yes	Yes	Yes	Yes	Some	Precise	Yes	Yes	Yes
Usami, 2011 [88]	Yes	Yes	Yes	cannot tell	Yes	Yes	Small	Not precise	Yes	Yes	Yes
Zhang, 2012 [89]	Yes	Yes	cannot tell	Yes	Yes	Yes	Some	Precise	Yes	Yes	Yes
Zhang, 2013 [90]	Yes	cannot tell	Yes	cannot tell	Yes	Yes	Some	Partial	Yes	Yes	Yes
Sadahiro, 2014 [57]	Yes	Yes	Yes	cannot tell	Yes	Yes	Some	Precise	Yes	Yes	Yes
Aisu, 2015 [91]	Yes	No	cannot tell	No	Yes	Yes	Some	Precise	Yes	Yes	Yes
Kotzampassi, 2015 [58]	Yes	Yes	Yes	Yes	Yes	Yes	Some	Precise	Yes	Yes	Yes
Mayes, 2015 [92]	Yes	Yes	Yes	cannot tell	Yes	Yes	Some	Precise	Yes	Yes	Yes
El-Ghazely, 2016 [93]	Yes	Yes	Yes	Yes	Yes	Yes	Some	Precise	Yes	Yes	Yes
Kotmatsu, 2016 [94]	Yes	Yes	Yes	No	No	Yes	Some	Precise	Yes	Yes	Yes
Yang, 2016 [95]	Yes	Yes	Yes	Yes	Yes	Yes	Small	Precise	Yes	Yes	Yes

1. Does the trial address a clearly focused issue? 2. Was the assignment of patients to treatments randomized? 3. Were all the patients who entered the trial properly accounted for at its conclusion? Were patients, health workers and study personnel “blind” to treatment? 5. Were the groups similar at the start of the trial? 6. Aside from the experimental intervention, where the groups treated equally? 7. How large was the treatment effect? 8. How precise was the estimate of the treatment effect? 9. Can the results be applied to local population, or in your context? 10. Were all clinically important outcomes considered? 11. Are the benefits worth the harms and costs? ^*∗*^NA-not applicable, because was no control group.

**Table 5:** CASP quality assessment checklist of included case study using the CASP checklist for appraising a case-controlled study.

First author, year	Section A							Section B			Section C	
	1	2	3	4	5	6a	6b	7	8	9	10	11
Thomson, 2012 [55]	Yes	Yes	Yes	No	cannot tell	No	cannot tell	Small	Mostly	Yes	Yes	Yes

1. Did the study address a clearly focused issue? 2. Did the authors use an appropriate method to answer their question? 3. Were the cases recruited in an acceptable way? 4. Were the controls selected in an acceptable way? 5. Was the exposure accurately measured to minimise bias? 6a. Aside from the experimental intervention, where the groups treated equally? 6b. Have the authors taken account of the potential confounding factors in the design and/or in their anaylsis? 7. How large was the treatment effect? 8. How precise was the estimate of the treatment effect? 9. Do you believe the results? 10. Can the results be applied to local population? 11. Do the results of this study fit with other available information?

**Table 6:** Most commonly used probiotic species in the investigated studies against wound pathogens.

Probiotic species	Study type		
	*In vitro*	Animal	Clinical study
	References	References	References
*Lactobacillus plantarum*	[61]^#^, [64, 67, 71, 74, 75]	[61]^#^, [76, 78–80]	[22, 58, 82, 84–87, 90]
*Lactobacillus casei*	[64, 71, 73]		[55, 56, 83, 88–90, 94]
*Lactobacillus acidophilus*	[70, 71, 73, 75]		[58, 87, 95]
*Lactobacillus rhamnosus*	[66, 69, 70, 73]		[90, 92]
*Lactobacillus fermentum*	[63, 65]	[77]	[93]
*Bifidobacterium breve*			[56, 83, 88, 94]
*Bifidobacterium longum*	[71]		[87, 88, 95]
*Lactobacillus reuteri*	[64, 66, 72]		
*Bifidobacterium lactis*	[71]		[58, 90]
*Bifidobacterium longum*	[71]		[87, 88, 95]
			[57]
*Bifidobacterium bifidum*	[75]		[93]
*Lactobacillus delbrueckii*	[71]		[84, 85]
*Pediococcus pentosaceus*			[84, 85]
*Leuconostoc mesenteroides*			
*Propionibacterium acnes*	[68]^#^	[68]^#^	
*Lactobacillus brevis*	[71]		[90]
*Lactobacillus paracasei*			[84, 85]
*Saccharomyces boulardii*			[58]
*Bifidobacterium animalis*	[71]		
*Lactobacillus salivarius*	[71]		
*Bacillus coagulans*	[75]		
*Bacillus mesentericus*			[91]
*Clostridium butyricum*			[91]

^#^Study includes *in vitro* and animal model studies.

## Supplementary Materials

Supplementary Materials The supplement file is a PRISMA checklist.
